# Remodelling landscape of tissue‐engineered bladder with porcine small intestine submucosa using single‐cell RNA sequencing

**DOI:** 10.1111/cpr.13343

**Published:** 2022-09-30

**Authors:** Liao Peng, Xi Jin, Qing He, Xiao‐shuai Gao, Wei Wang, Xiao Zeng, Hong Shen, De‐yi Luo

**Affiliations:** ^1^ Department of Urology Institute of Urology, West China Hospital, Sichuan University Chengdu PR China; ^2^ Department of Urology The Third People's Hospital of Chengdu Chengdu PR China

## Abstract

**Objective:**

Bioscaffolds are widely used for tissue engineering, but failed and inconsistent preclinical results have hampered the clinical use of bioscaffolds for tissue engineering. We aimed to construct a cellular remodelling landscape and to identify the key cell subpopulations and important genes driving bladder remodelling.

**Methods:**

Twenty‐four reconstructed mouse bladders using porcine small intestinal submucosa (PSIS) were harvested at 1, 3, and 6 weeks to perform single‐cell RNA sequencing. Cell types were identified and their differentially expressed genes (DEGs) at each stage were used for functional analysis. Immunofluorescence was used to validate the specific cell type.

**Results:**

The remodelling landscape included 13 cell types. Among them, fibroblasts, smooth muscle cells (SMCs), endothelial cells, and macrophages had the most communications with other cells. In the process of regeneration, DEGs of fibroblasts at 1, 3, and 6 weeks were mainly involved in wound healing, extracellular matrix organization, and regulation of development growth, respectively. Among these cells, Saa3^+^ fibroblasts might mediate tissue remodelling. The DEGs of SMCs at 1, 3, and 6 weeks were mainly involved in the inflammatory response, muscle cell proliferation, and mesenchyme development, respectively. Moreover, we found that Notch3^+^ SMCs potentially modulated contractility. From 1 to 6 weeks, synchronous development of endothelial cells was observed by trajectory analysis.

**Conclusions:**

A remoulding landscape was successfully constructed and findings might help surficial modifications of PSIS and find a better alternative. However, more in vivo and in vitro studies are needed to further validate these results.

## Graphical abstract

Bioscaffolds are widely used for tissue engineering, but failed and inconsistent preclinical results have hampered the clinical use of bioscaffolds for tissue engineering. We performed single‐cell RNA sequencing to decipher a cellular remodelling landscape and to identify the key cell subpopulations and important genes driving bladder remodelling. A total of 13 cell types were identified during the bladder regeneration process. Among them, Saa3^+^ fibroblasts mainly mediated tissue remodelling, Notch3^+^ SMCs might modulate contractility, endothelial cells developed synchronously, and M2‐like macrophages balanced inflammation responses. We constructed bladder regeneration landscape using PSIS, investigated the cell–cell interactions, and highlighted the key genes and signalling pathways involving in this process. These findings may help surficial modifications of PSIS and find a better alternative of PSIS.

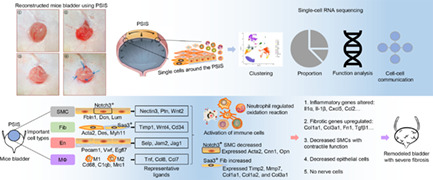

## 1 INTRODUCTION

Although tissue engineering has been widely and successfully used in the areas of urology, vascular surgery, cardiac surgery, neurosurgery, and orthopaedic surgery, general challenges, such as bioscaffold exposure, infection, rejection, and fibrosis, urgently needed to be resolved.^1^, ^2^, ^3^ In the past two decades, various advantageous scaffold materials have been developed, seed cells with special functions have been planted, and several growth‐promoting factors have been carried, but the complete wound healing, neurolization, vascularization, and functional recovery remain major challenges.^4^, ^5^, ^6^

In the field of urology, bowel segment grafting for cystoplasty is needed if a patient had severe bladder fibrosis or low‐compliance bladder, or needed cystectomies.^7^ However, the use of gastrointestinal segments may lead to adverse events, including nutritional alterations, peritoneal adhesions, abscesses, enteric fistulae, excessive mucus production, bladder rupture, bacterial colonization, stone formation, or malignancy.^8^, ^9^, ^10^ To minimize these complications, the field of tissue engineering attempts to develop alternative biological scaffolds to replace the gastrointestinal segment. In 2006, Atala et al. first reported that tissue engineering techniques can be used to generate bladders that can be implanted in patients requiring cystoplasty.^11^ Subsequently, researchers have gradually focused on scaffold materials and functionality restoration in bladder tissue engineering.^6^, ^9^, ^10^

Among promising biomaterials, porcine small intestinal submucosa (PSIS) is one of the most thoroughly studied scaffolds for urinary tract reconstruction in both experimental and preclinical models.^6^, ^12^ PSIS is a xenogeneic, acellular, biocompatible, biodegradable, and native collagen‐based bioscaffold that is optimal for bladder regeneration without cell seeding in various animal models and clinical trials.^6^ Although the findings suggested that PSIS supported bladder cell growth, and PSIS‐regenerated bladders were histologically and functionally indistinguishable from normal functional tissues, clinical utilization of PSIS for bladder augmentation has been hampered by inconsistent preclinical results.^6^, ^13^, ^14^ Specifically, severe immune responses and fibrosis, and the difficulty in neurolization and vascularization may lead to a nonfunctional bladder. However, these issues are challenging and difficult to address given that the molecular mechanism driving bladder regeneration is unclear.

Single‐cell RNA sequencing (scRNA‐seq) is capable of deciphering the cell types involving in the bladder regeneration process, identifying specific cell subsets with high expression level of fibrotic genes, and predicting key signalling pathways driving the proliferation and development of nerve and vascular cells.^15^ Thus, we applied scRNA‐seq to construct a cellular regeneration landscape in mice that underwent augmentation cystoplasty with PSIS and to better understand the regenerative mechanism in the remodelling process. The findings will provide insights and promising targets for developing more suitable biomaterials for bladder tissue regeneration.

## 2 MATERIALS AND METHODS

### 2.1 Mice

The animal experiments were approved by the Animal Research Ethics Committee of West China Hospital, Sichuan University (No. 2019270A). Eight‐week‐old male C57BL/6 mice were purchased from Dossy Experimental Animals Co., Ltd. (Chengdu, China) and maintained in the specific pathogen‐free animal research centre. Twenty‐four mice were randomly divided into 4 groups: week 1 postoperatively (*n* = 6), week 3 postoperatively (*n* = 6), week 6 postoperatively (*n* = 6), and the sham group (week 6, *n* = 6). Then, augmentation cystoplasty was performed in the surgery group.

### 2.2 Augmentation cystoplasty (AC) using porcine small intestinal submucosa (PSIS)

**FIGURE 1:**
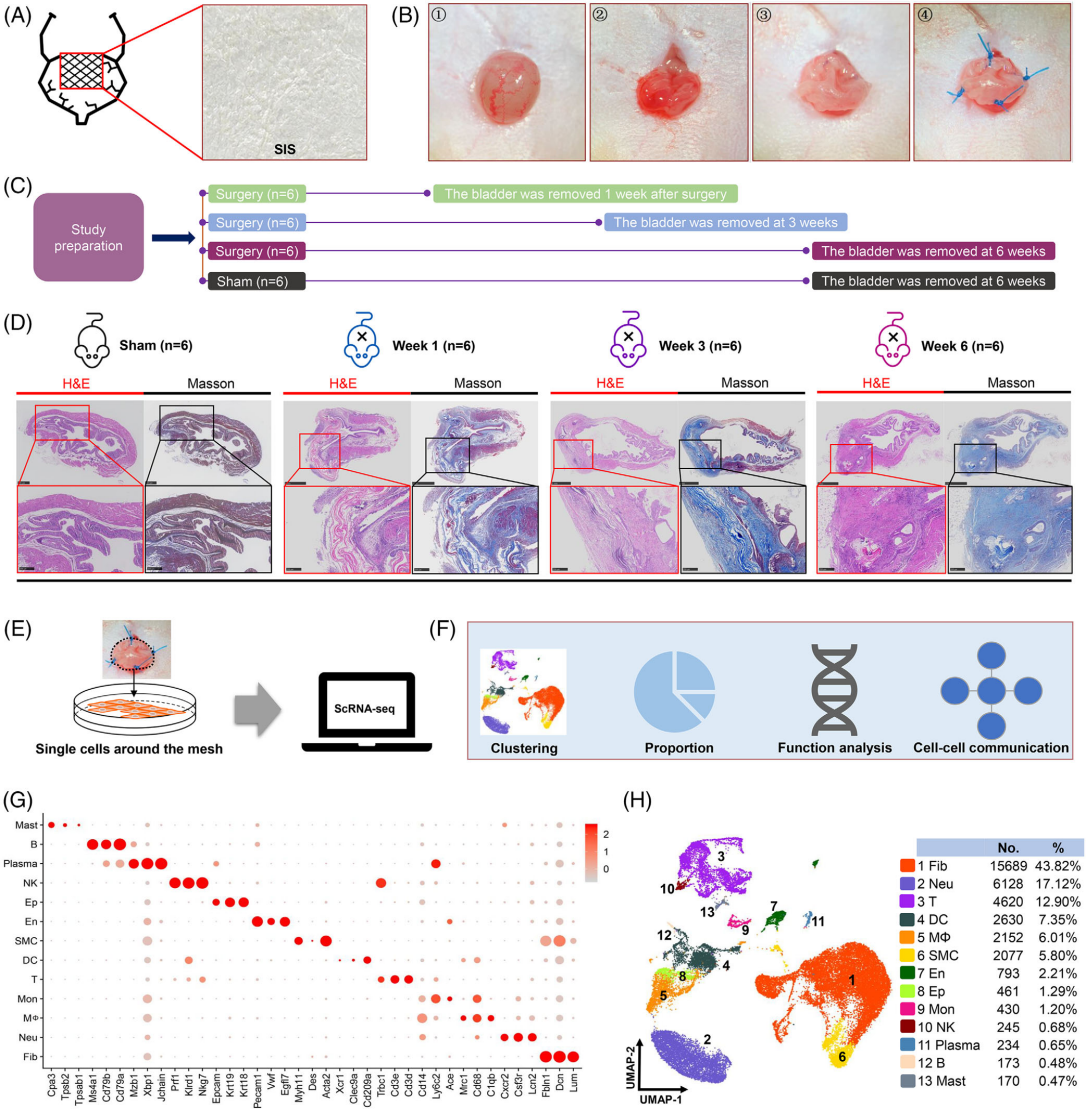
Single‐cell remodelling landscape of tissue‐engineered bladders. (A) The PSIS scaffold was used to perform augmentation cystoplasty. (B) Step‐by‐step cystoplasty on 18 mouse bladders. (C) The mouse bladders were harvested post cystoplasty at 1, 3, and 6 weeks. (D) H&E and Masson staining of bladder sections were performed. (E) PSIS‐wrapped partial bladder tissues were used for single‐cell RNA sequencing. (F) Cell clustering and functional investigations were performed. (G) Canonical marker genes were included to identify specific cell types. (H) Thirteen cell subpopulations were identified in the constructed landscape. B, B cell; DC, dendritic cell; En, endothelial cell; Ep, epithelial cell; Fib, fibroblast; MΦ, macrophage; Mast, mast cell; Mon, monocyte; Neu, neutrophil; NK, natural killer cell; Plasma, plasma cell; PSIS, porcine small intestinal submucosa; SMC, smooth muscle cell; T cells.

All procedures were performed by one researcher (Figure 1A,B). AC using PSIS (Biodesign® COOK Medical, C‐SLH‐45‐4x7, West Lafayette, IN, USA) was performed according to previous surgical methods with slight modifications.^16^, ^17^ Mice were anaesthetised by isoflurane inhalation. In the supine position, lower abdominal shaving was conducted to expose the surgical area, and the skin was prepared in a sterile fashion. A 0.5–1 cm low midline abdominal incision was made, and the peritoneal cavity was entered. A 4 mm incision was created on the bladder dome, and a tailored 25 mm^2^ square section of 4‐layer PSIS was sutured to the native bladder with 8–0 Vicryl (Ethicon, Somerville, NJ) in a watertight fashion. Subsequently, permanent sutures were placed to identify PSIS scaffolds from host bladder tissues at harvest. Then, bladders were infused with 10 ml saline to test a watertight closure. The abdominal wall and skin were closed sequentially, and animals were then quarantined. In sham‐operated controls, mice were subjected to midline abdominal incision followed by abdominal wall and skin closure without performing incision or bladder augmentation. After surgery, the mice were individually housed in conventional cages and monitored daily.

To perform histological staining and gene sequencing, the mice were sacrificed, and their bladders were harvested post AC at weeks 1, 3, and 6 (Figure 1C,D).

### 2.3 Histologic staining

Mouse bladder were harvested and fixed in fresh 4% paraformaldehyde for 24 h, embedded in paraffin, cut into 4‐μm‐thick sections, and mounted on slides. The slides were stained with haematoxylin and eosin (H&E) to present cell distribution or stained with Masson's trichrome to show collagen deposition in each group. Images from each section were captured by a digital camera (Olympus Corporation, Tokyo, Japan).

### 2.4 Bladder procurement and processing

In each group, 6 mouse bladders were harvested after the mice were euthanized. The partial bladder tissues wrapping PSIS were then cut into pieces and enzymatically digested with collagenase type 1 (1 mg/mL; 17,100,017) and DNase I (0.2 mg/mL; 10,104,159,001) for 20 min at 37°C with agitation. Then, we applied StemPro Accutase Cell Dissociation Reagent (A1110501; Gibco) to dissect the sticky clumps of cells for 10 min and then terminated digestion with DPBS. Subsequently, we removed red blood cells using RBC lysis buffer (B250015; BioLegend) for 5 min and then centrifuged them at 300*g* for 5 min at 4°C. After discarding the supernatant, the cells were resuspended in DPBS at 4°C and sieved through a 40‐μm cell strainer. Finally, viability assessment and cell counting were performed, and a viability over 90% was considered optimal for downstream analysis.

### 2.5 Single cell library preparation and sequencing

Single‐cell RNA‐seq libraries were constructed with Chromium Single‐cell 3' Reagent Kits (v3) based on the manufacturer's protocol. A single‐cell suspension was loaded on the Chromium Single Cell Controller Instrument (10X Genomics) to generate single‐cell gel beads in emulsions (GEMs). After GEMs were normally formed, reverse transcription reactions engaged barcoded full‐length cDNA followed by the disruption of emulsions using the recovery agent and cDNA clean up with DynaBeads Myone Silane Beads (Thermo Fisher Scientific). cDNA amplification was performed by PCR. Subsequently, the amplified cDNA was fragmented, end‐repaired, A‐tailed, index adaptor ligated and library amplified. Finally, these libraries were sequenced on the Illumina sequencing platform (HiSeq X Ten), and 150 bp paired‐end reads were generated (CapitalBio Technology, Beijing).

### 2.6 Data preprocessing and quality control

Preliminary sequencing results were converted to FASTQ files by CellRanger (v3.1.0). The sequenced data were then aligned to the mouse genome reference sequence (mm10). Cell Ranger was also applied for preliminary data analysis and generated a web‐based file containing basic sequencing data (Table S1). Then, the matrix was converted into a Seurat object by the R package Seurat (v3.1.2).^15^ Cells with gene numbers <200 or >10,000 or with >10% mitochondrial‐derived UMI counts were considered low‐quality cells and removed. In addition, the mitochondrial genes and ribosomal genes were also removed from the gene expression matrix. Finally, a total of 35,802 high‐quality cells were included for map investigation.

### 2.7 Visualization and cell clustering

Batch differences were considered and removed given that the sequenced data were obtained at different stages per time setting. Then, the top 2000 variable genes were selected by FindVariableFeautres for principal component analysis (PCA). Cells were separated into 13 clusters by the FindClusters function using the top 20 principal components. The uniform manifold approximation and projection (UMAP) algorithm was applied to visualize cells in a two‐dimensional space. Conventional markers described in a previous study were used to categorize each cell into a known biological cell type,^15^, ^18^, ^19^ including fibroblasts (Fbln1, Dcn, and Lum), smooth muscle cells (Myh11, Des, and Acta2), endothelial cells (Pecam1, Vwf, and Egfl7), epithelial cells (Epcam, Krt18, and Krt19), T cells (Cd3d, Cd3e, and Trbc1), B cells (Ms4a1, Cd79a, and Cd79b), neutrophils (Cxcr2, Csf3r, and Lcn2), dendritic cells (Cd209a, Clec9a, and Xcr1), monocytes (Cd14, Ly6c2, and Ace), macrophages (Cd68, C1qb, and Mrc1), natural killer cells (Nkg7, Klrd1, and Prf1), plasma cells (Mzb1, Xbp1, and Jchain), and mast cells (Cpa3, Tpsb2, and Tpsab1). Subsequently, major cell types were further clustered into subclusters to detect heterogeneity within each cell type.

### 2.8 Function analysis of differently expressed genes (DEGs)

Genes expressed in more than 10% of the cells in a cluster and with an average log (fold change) of greater than 0.5 were selected as DEGs by the Seurat FindMarkers function. Furthermore, the DEGs between the two selected groups were identified. The false‐positive result was corrected by the adjusted *p* value (adj. *p* value) using the Benjamini–Hochberg method. The “adj. *p* value <0.05” and “|logFC| > 0.5” were set as the cut‐off criteria. Gene Ontology (GO) enrichment analysis and Kyoto Encyclopedia of Genes and Genomes (KEGG) pathway analysis were performed on DEGs with a Bonferroni‐corrected *p* value of <0.05.

### 2.9 Cell–cell interaction analysis

Cell–cell interactions between the selected cell type and residual cell types were predicted based on known ligand–receptor pairs by Cellphone DB.^20^ Interaction pairs with a *p* value of <0.05 were returned and used to evaluate links between cell types. The mouse genes were transformed to human genes using Biomart.

### 2.10 Pseudotime trajectory analysis by monocle2

The developmental trajectory of subclustered cell types was predicted by monocle2.^21^ Significantly changed genes were identified by the differential GeneTest function in Monocle2 with a *q*‐value <0.01.

### 2.11 Immunofluorescence staining (IF)

IF was performed to validate the specific cell types. The sections originating from mouse bladders of each group were labelled with primary antibodies, including anti‐Cd163 (1:100; Abcam, ab182422) and anti‐Cd31 (1:100; Abcalonal, A2104), both followed by 488 goat anti‐rabbit FITC‐conjugated IgG secondary antibodies (Servicebio, GB22303). Then, both anti‐Fbln1 (1:100; Novus, NBP252918) and anti‐Saa3 (1:100, Abcam, ab282730) antibodies and anti‐Acta2 (1:200; Novus, NBP2‐22120) and anti‐Notch3 (1:200; Abcam, ab23426) antibodies were added, followed by 488 goat anti‐rabbit FITC‐conjugated IgG secondary antibodies (Servicebio, GB22303) and 568 goat anti‐mouse Cy3‐conjugated IgG secondary antibodies (Servicebio, GB21301). In addition, nuclei were labelled with 4′6‐diamidino‐2‐phenylindole (DAPI, Sigma–Aldrich Co., Ltd). All IF images were captured by a digital camera (3DHISTECH, Case Viewer 2.4).

### 2.12 Statistical analysis

The statistical methods were detailly described in each step. Statistical analysis was performed with R software (v3.6.1). A *p* value <0.05 was considered statistically significant.

## 3 RESULTS

### 3.1 Single‐cell remodelling landscape of tissue engineered bladder

To establish a cell map of tissue regeneration after AC using PSIS, scRNA‐seq was applied to investigate the cell subsets and their molecular functions in dynamically growing mouse bladders. After bladder reconstruction with PSIS was performed (Figure 1A,B), mouse bladders were harvested after AC at 1, 3, and 6 weeks (Figure 1C). Images of H&E and Masson staining showed that PSIS scaffolds degraded gradually, and collagen deposition increased gradually during the tissue repair process (Figure 1D). Then, the partial bladder tissues wrapping the PSIS were enzymatically digested and subsequently used for scRNA‐seq (Figure 1E,F). After data normalization and quality control, 35,802 high‐quality single cells were loaded for downstream analysis (Figure S1A, Table S1). Thirteen cell types were identified using canonical marker genes (Figures 1G, S1B), which included 53.13% nonimmune cells (fibroblasts, smooth muscle cells (SMCs), endothelial cells (Ens), and epithelial cells) and 46.87% immune cells (neutrophils, T cells, dendritic cells, macrophages, monocytes, natural killer cells, plasma cells, B cells, and mast cells) (Figure 1H). The distribution of cells at each stage indicated that immune cells decreased and bladder scaffold cells increased gradually during the tissue regeneration process, and they returned to a normal level over time, as in the sham group (Figure S1C, Table S2). The representative differentially expressed genes (DEGs) in each cluster were identified and used for enrichment analysis. Each cell type plays an important and unique role in bladder regeneration. For example, fibroblasts mainly mediate extracellular matrix remodelling, and neutrophils regulate the immune response (Figure S1D). These findings overall depicted a cell landscape in bladder tissue engineering using PSIS.

### 3.2 Fibroblasts, SMCs, Ens, and macrophages play important roles in bladder regeneration

**FIGURE 2:**
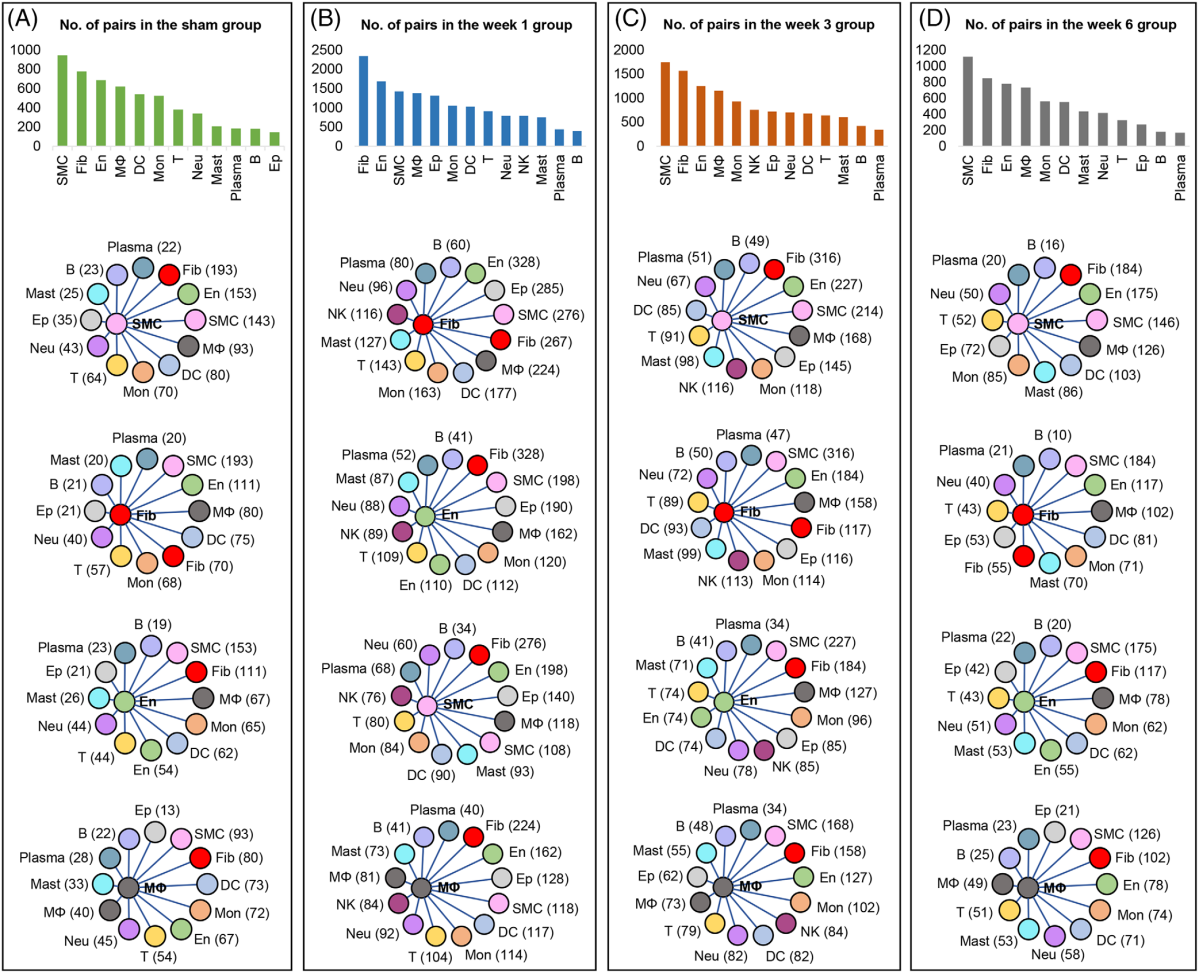
Cell–cell communications in the constructed landscape. (A) The number of interaction pairs in the sham group and the links between SMCs, Fib, En, and MΦs with other cell types. (B) The number of interaction pairs in the week 1 group and the links between SMCs, Fib, En, and MΦs and other cell types. (C) The number of interaction pairs in the week 3 group and the links between SMCs, Fib, En, and MΦs and other cell types. (D) The number of interaction pairs in the week 6 group and the links between SMCs, Fib, En, and MΦs and other cell types. B, B cell; DC, dendritic cell; En, endothelial cell; Ep, epithelial cell; Fib, fibroblast; MΦ, macrophage; Mast, mast cell; Mon, monocyte; Neu, neutrophil; NK, natural killer cell; Plasma, plasma cell; SMC, smooth muscle cell; T cells.

Cell–cell communication mediated by ligand–receptor complexes is critical to coordinating diverse biological processes, such as development, differentiation, and inflammation.^20^ Using Cellphone DB analysis, the number of interaction pairs at each stage was calculated. Interestingly, fibroblasts, SMCs, Ens, and macrophages had the most communication with other cells at each stage (Figure 2). This analysis also identified both condition‐specific and cell type‐specific communications. Compared with the sham operation group, these four cell types in the week 1 group had the most interaction pairs, followed by the week 3 group. Then, the number of interactions across the four cell subsets gradually returned to a normal level over time (Figure 2). Regarding cell–cell communication across the four cell types, we found that each cell type uniquely expressed receptors and ligands at each stage. For example, fibroblasts mainly contacted other cells via the ligands Timp1, Cd34, Wnt2, and Rarres2 in the week 1, week 3, week 6, and sham groups, respectively (Figure S2). Then, the top 30 interaction pairs at each stage were used to perform functional enrichment analysis (Table S3). The differentially expressed ligands and receptors were mainly enriched in cell development and neuron differentiation in the week 1 group, immune response, and tissue morphology in the week 3 group, cell migration in the week 6 group, and tissue homeostasis in the sham group (Figure S3), suggesting that cell–cell interactions play an important role in bladder tissue engineering. Subsequently, subclustering of these four cell types was performed (Table S4).

### 3.3 Saa3^+^ fibroblasts might mediate tissue remodelling

**FIGURE 3:**
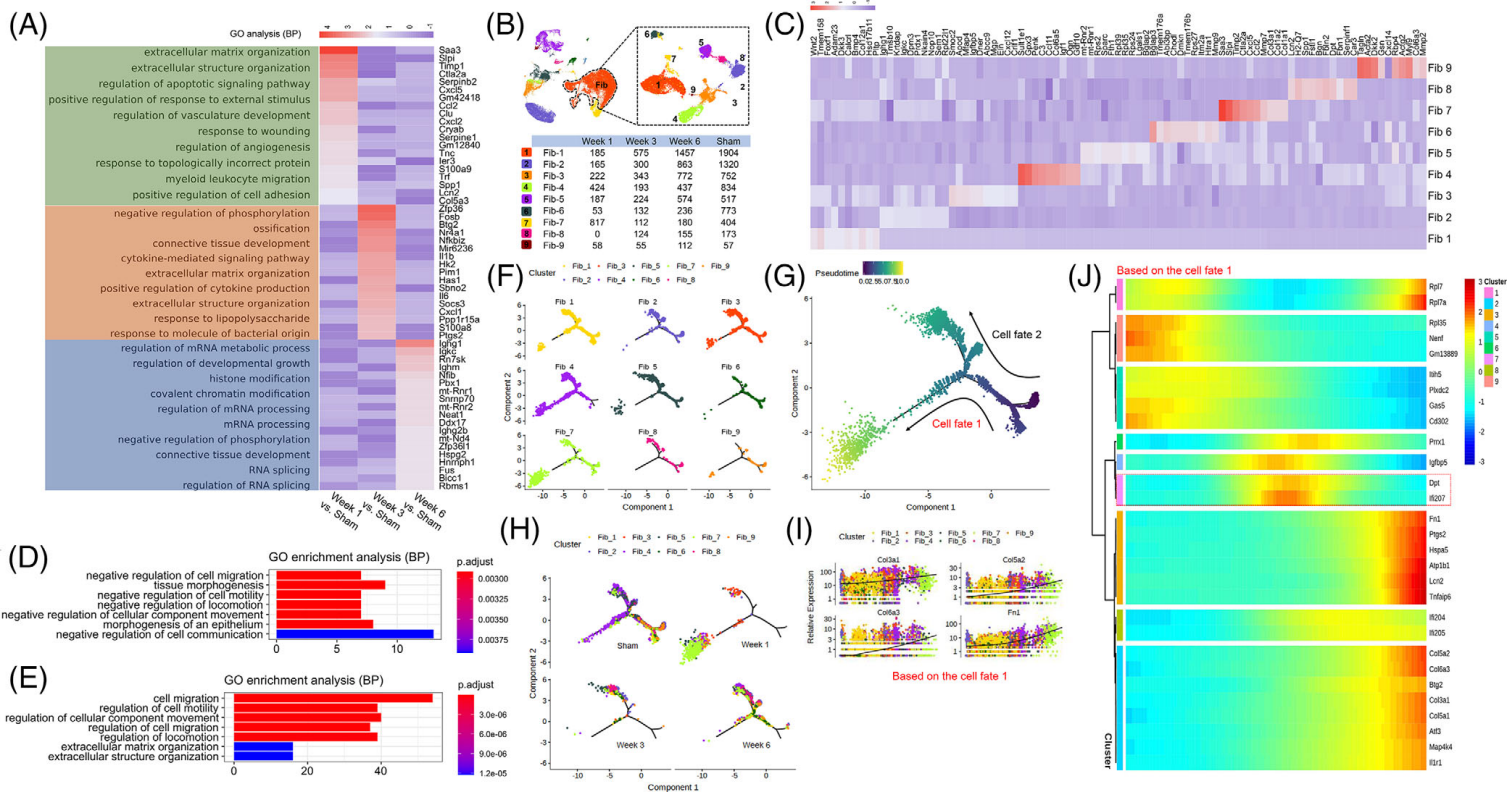
Focused analysis of fibroblasts revealed Saa3^+^ fibroblasts. (A) Enrichment analysis (biological process) of DEGs of fibroblasts at each stage obtained by comparison with the sham group. (B) Nine clusters of fibroblasts were identified by reclustering. (C) The top 10 DEGs of each subfibroblast. (D) Enrichment analysis (biological process) of DEGs of fibroblast‐1 cells. (E) Enrichment analysis (biological process) of DEGs of fibroblast‐7 cells. Pseudotime trajectory analysis of subfibroblasts by cluster (F), pseudotime (G), and group (H). (I) The expression of genes related to collagen deposition gradually increased during the tissue repair process (cell fate 1). (J) Key genes drive the developmental direction of subfibroblasts (cell fate 1). DEG, differentially expressed gene; Fib, fibroblast.

**FIGURE 4:**
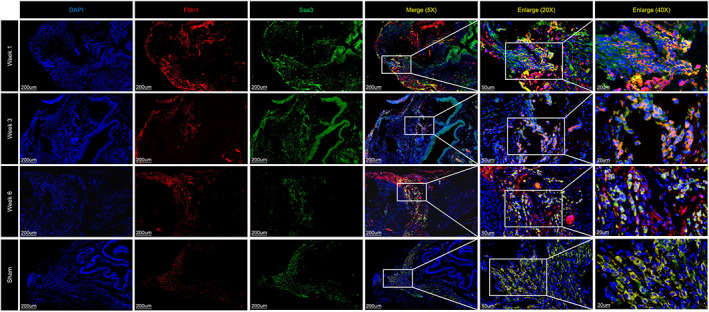
Immunofluorescence staining revealed the distribution of Saa3^+^ fibroblasts at each stage.

Compared with the sham group, the DEGs of fibroblasts at 1, 3, and 6 weeks were mainly involved in wound healing, extracellular matrix organization, and regulation of developmental growth (Figure 3A). Reclustering of fibroblasts identified 9 subpopulations (Figure 3B,C). Of note, fibroblast‐1 was the main cell type in the week 3, week 6, and sham groups, but fibroblast‐7 was the main cell type in the week 1 group (Figure 3B). The DEGs of fibroblast‐1 were mainly related to the negative regulation of cell migration (Figure 3D). However, the DEGs of fibroblast‐7 showed that these cells participated in cell migration and remodelling of the extracellular matrix (Figure 3E), indicating that there may be an interaction between fibroblast‐1 and fibroblast‐7. The high expression levels of Timp2, Mmp7, Col1a1, Col1a2, and Col3a1 in fibroblast‐7 cells also suggested that they were the main cells involved in tissue repair and organ morphology^22^ (Figure 3C). Pseudotime trajectory analysis based on the cell subset, pseudotime, and cluster was then performed to establish a developmental trajectory of 9 clusters of fibroblasts over time (Figure 3F,G). Two specific cell fates were observed across subfibroblasts (Figure 3G). Both two developmental directions began with the most fibroblasts in the sham group, but cell fate 1 ended up with the fibroblasts in the week 1 group, which were mainly composed of Fibroblast‐7 cells (Figure 3E,H). However, cell fate 2 ended up with the major fibroblasts in the sham and week 6 groups. According to the functional analysis, fibroblast‐1 and fibroblast‐7 were most related to the bladder remodelling. Intriguingly, the developmental trajectory from fibroblast‐1 to fibroblast‐7 was in line with the cell fate 1 direction. Thus, the cells in the cell fate 1 were captured to perform downstream analysis. The expression of genes related to collagen deposition in the cell fate 1 gradually increased during the tissue repair process (Figure 3I). Dpt and Ifi207 may be the key genes guiding the functional differentiation of fibroblast‐7 cells (Figure 3J). Given that Saa3 was only expressed in fibroblast‐7 cells, this cluster was defined as Saa3^+^ fibroblasts (Figure 3C). To confirm the location of Saa3^+^ fibroblasts in regenerated bladders, IF was performed, and the results demonstrated the existence of this cell type (Figure 4).

### 3.4 Notch3^+^ SMCs might modulate contractility

**FIGURE 5:**
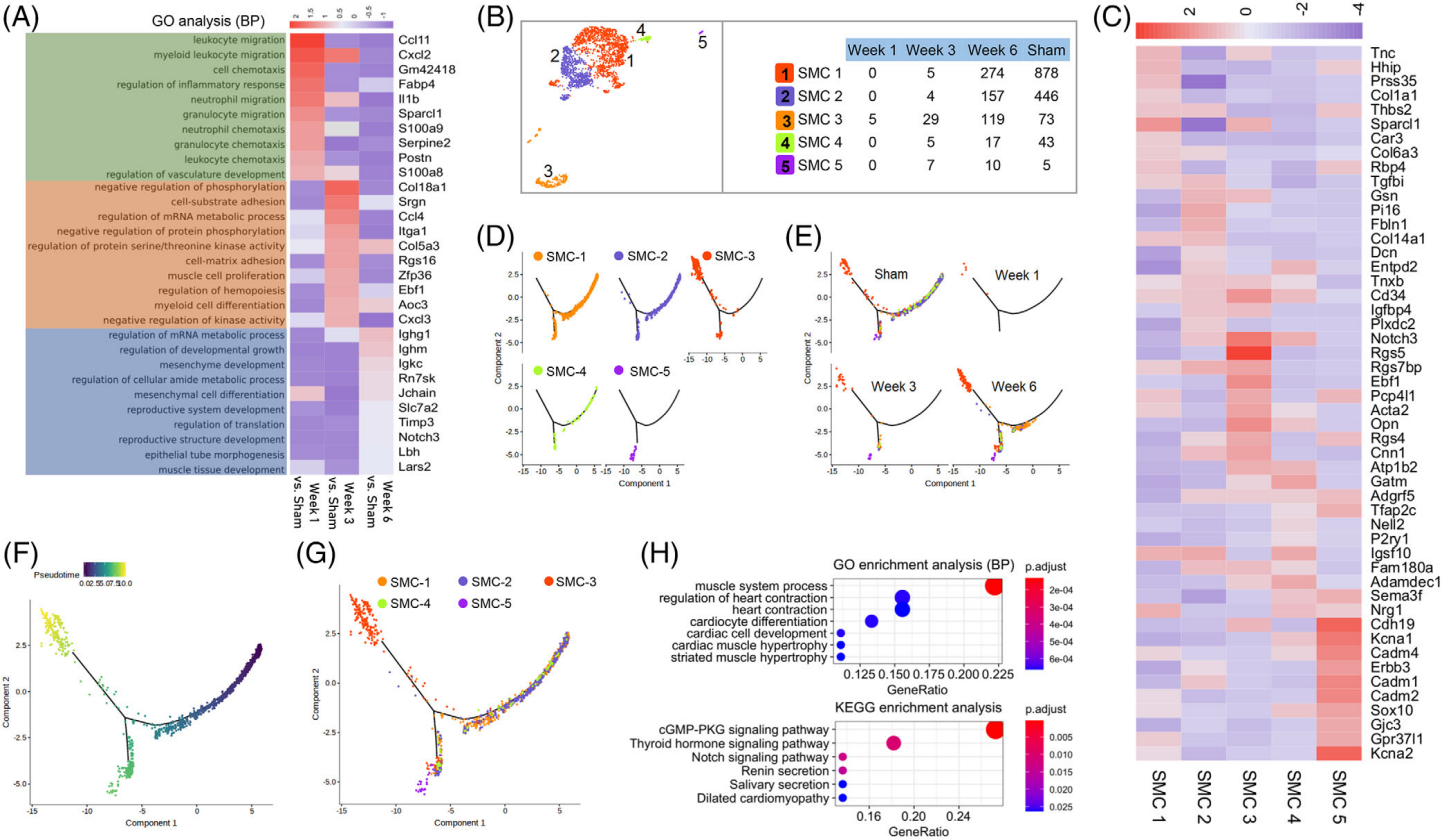
Focused analysis of SMCs revealed Notch3^+^ SMCs. (A) Enrichment analysis (biological process) of DEGs of SMCs at each stage obtained by comparing the sham group. (B) Five clusters of SMCs were identified by reclustering. (C) The top 10 DEGs of each sub‐SMC. Pseudotime trajectory analysis of sub‐SMCs by cluster (D), group (E), pseudotime (F), and distribution (G). (H) Enrichment analysis (biological process) of DEGs of SMCs‐3. DEG, differentially expressed gene; SMC, smooth muscle cell.

**FIGURE 6:**
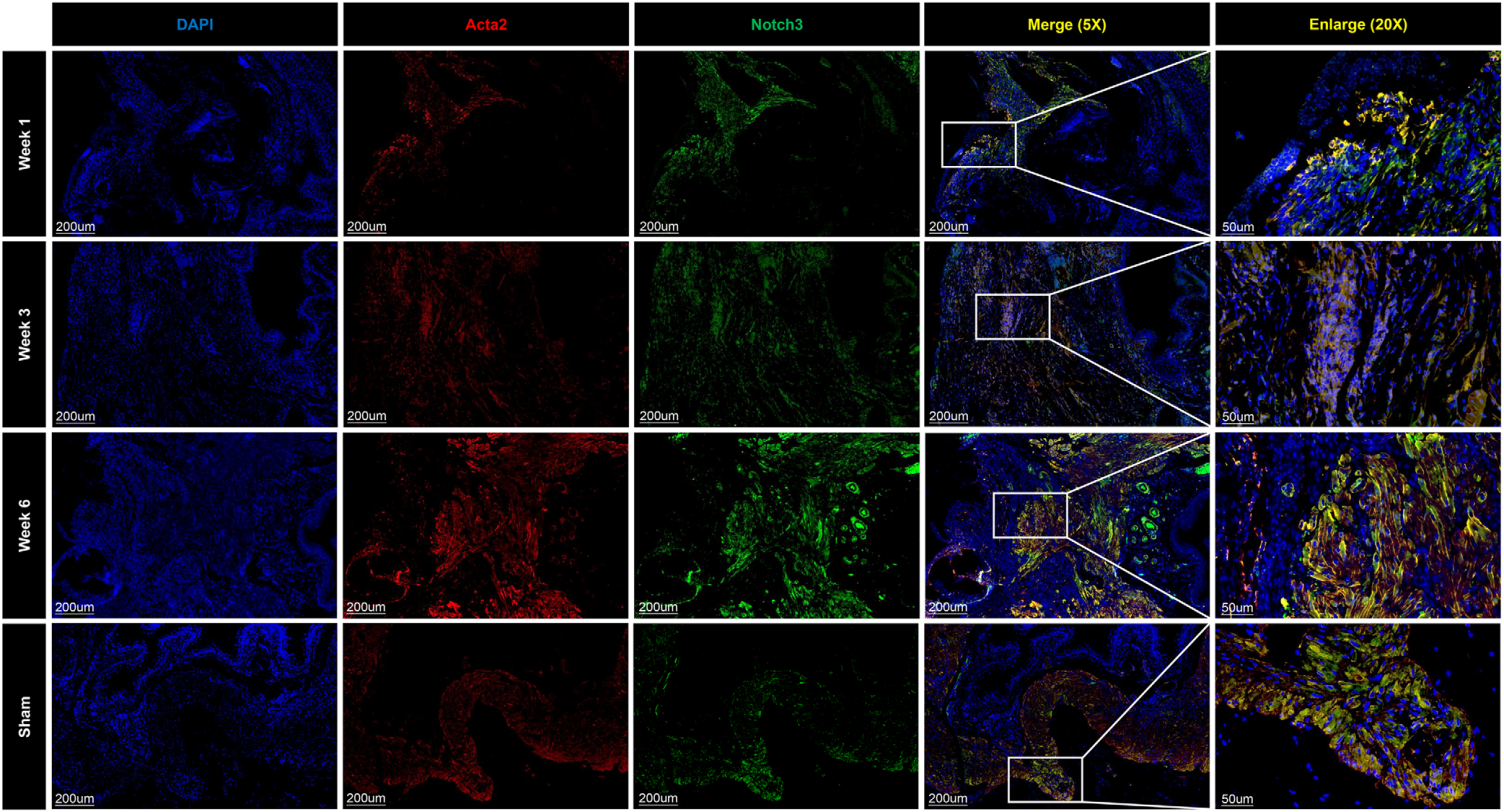
Immunofluorescence staining revealed the distribution of Notch3^+^ smooth muscle cells at each stage.

Compared with the sham group, the DEGs of SMCs at 1, 3, and 6 weeks were mainly involved in the inflammatory response, muscle cell proliferation, and mesenchymal development (Figure 5A). Five clusters of SMCs were identified after subclustering (Figure 5B), but it should be noted that the number of SMCs is small and the regeneration of SMCs was relatively difficult. The heatmap shows the top 10 DEGs of each new cluster (Figure 5C). Pseudotime trajectory analysis demonstrated that SMC‐3 cells were mainly distributed in the terminal stage of the developmental trajectory (Figure 5D,G). With the expression of DEGs, such as Notch3, Acta2, Cnn1, and Opn in SMCs‐3, we found that the function of SMCs‐3 was mainly related to muscle system processes and muscle contraction (Figure 5H). Finally, the cluster was named as Notch3^+^ SMCs and their distributions in each stage were detected by IF (Figure 6).

### 3.5 Vascularization after AC with PSIS

**FIGURE 7:**
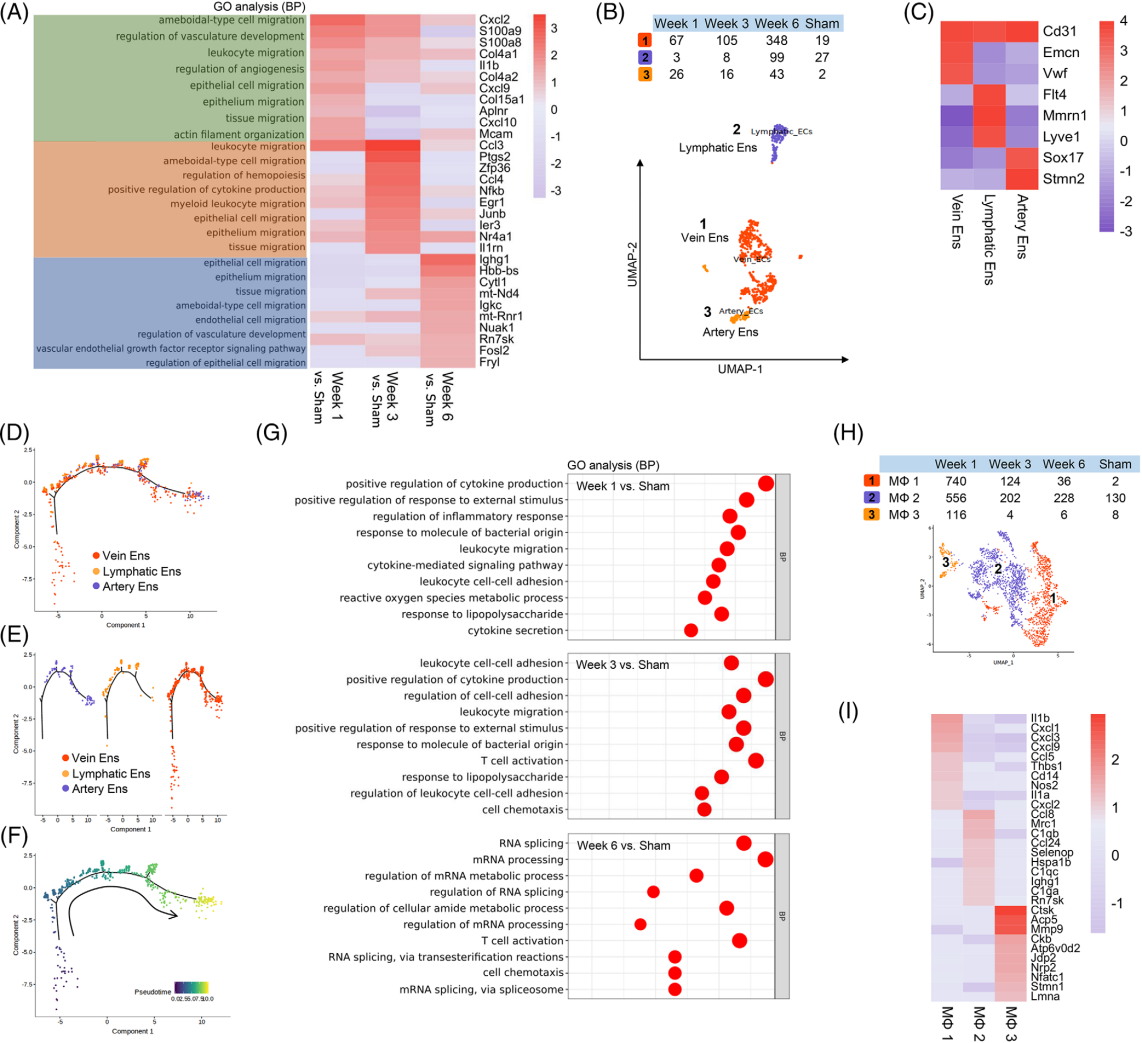
Focused analysis of Ens and MΦ. (A) Enrichment analysis (biological process) of DEGs of Ens at each stage obtained by comparison with the sham group. (B) Three clusters of Ens were identified by reclustering. (C) The top 10 DEGs of each sub‐En. Pseudotime trajectory analysis of sub‐Ens by distribution (D), group (E), and pseudotime (F). (G) Enrichment analysis (biological process) of DEGs of MΦs at each stage obtained by comparison with the sham group. (H) Three clusters of MΦs were identified by reclustering. (I) The top 10 DEGs of each sub‐MΦ. DEG, differentially expressed gene; MΦ, macrophages.

**FIGURE 8:**
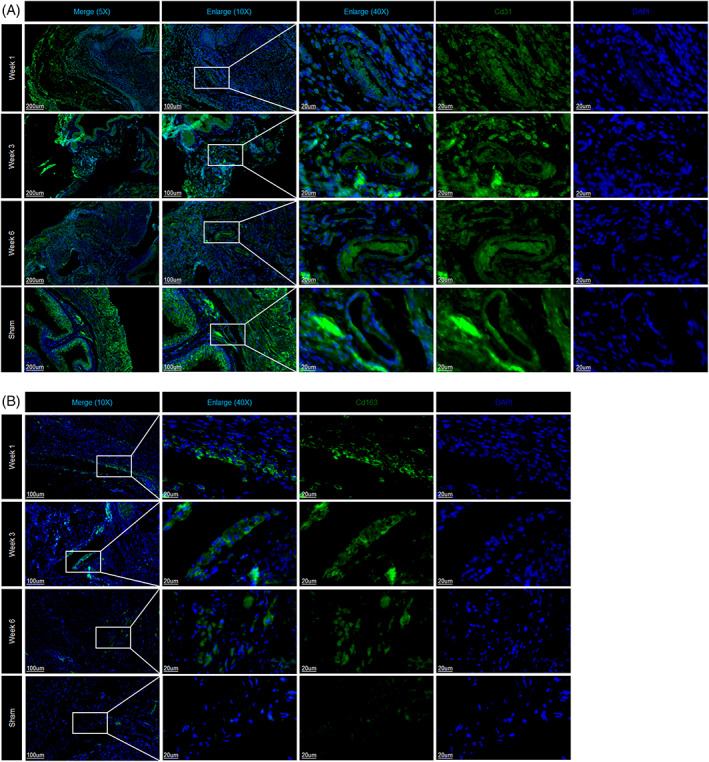
(A) Immunofluorescence staining revealed the distribution of endothelial cells at each stage. (B) Immunofluorescence staining revealed the distribution of macrophages at each stage.

After AC at 1 week, the function of DEGs in endothelial cells (Ens) was enriched in angiogenesis. Then, angiogenesis drove the migration of immune and nonimmune cells. At 6 weeks, vascular production guided tissue development (Figure 7A). Focused analysis of Ens identified vein Ens (Emcn and Vwf), artery Ens (Sxo17 and Stmn2), and lymphatic Ens (Flt4, Mmrn1, and Lyve1) (Figure 7B,C). Beginning with vein endothelial cells, then, synchronous development of all Ens was observed by trajectory analysis (Figure 7D–F), suggesting that vascularization post AC directly affects cell migration and tissue regeneration. Similarly, angiogenesis was also noted by IF (Figure 8A).

### 3.6 M2‐like macrophages mediated inflammation balance during bladder remodelling

Macrophages are key components of tissue repair and remodelling that occur during wound healing and allergies.^23^ At 1 and 3 weeks postsurgery, macrophages positively regulated the inflammatory response and mediated cell migration and adhesion (Figure 7G). Focused analysis of macrophages identified 3 clusters of subsets, including inflammatory macrophages (MΦ 1: Il‐1β, Il‐1a, Cxcl1, Cxcl3, Cxcl9, and Ccl5), M2‐like macrophages (MΦ 2: Mrc1, C1qb, and C1qc), and remodelling‐related macrophages (MΦ 3: Mmp9) (Figure 7H,I). M2‐like macrophages antagonize inflammation through phagocytotic and regulatory receptor‐mediated biological functions.^23^ M2‐like macrophages were evenly distributed postoperative at week 1, 3, and 6 (Figure 7H), implying their important role in inflammation balance. The gradually deceased number of inflammatory macrophages over time was consistent with our hypothesis (Figure 7H). Finally, M2‐like macrophages were confirmed by IF (Figure 8B).

### 3.7 Focused analysis of epithelial cells, neutrophils, and T cells during bladder regeneration

Epithelial cells (Eps) promoted wound healing at an early stage, but they were involved in tissue development and growth via the Wnt signalling pathway at 6 weeks^24^ (Figure S4A). According to the trajectory analysis, Eps‐3 was the most abundant cell type responding to stimuli via the HIF‐1 signalling pathway (Figure S4B–H). However, only a small number of Eps were captured, and future studies are needed to verify the current finding. Neutrophils were the largest number of immune cells. Neutrophil‐2 was mainly distributed at the end of the developmental trajectory (Figure S4I–M). Instead of promoting inflammation across immune cells, their roles were mainly related to regulating oxidation reactions (Figure S4M). Finally, we found that the functions of T cells were mainly enriched in self‐differentiation and development (Figure S5A). The reclustering of T cells demonstrated the diversity of T cells in tissue remodelling (Figure S5B–G). Regulatory T cells (Tregs), which contribute to inflammatory balance, were also identified. Proliferation T cells were located at the beginning of the developmental trajectory, and enrichment analysis revealed their functions in cell cycle processes and mitotic cell cycle processes (Figure S5H).

## 4 DISCUSSION

Patients with low‐compliance bladders resulting from various etiologies might require surgical reconstruction to increase bladder compliance and improve kidney function.^7^ PSIS is a commonly used bioscaffold for the bladder augmentation, but the severe immune response, fibrosis, and nonfunctional bladder could be observed after reconstructive surgery. These concerns are challenging due to a limited understanding of regenerative mechanism. Thus, understanding the mechanisms of regenerative processes will be helpful to monitor whether scaffold fabrication is desirable to support appropriate cell physiology, whether exogenously added biologically active agents lead to appropriate regeneration process, and whether progenitor cells differentiate into target cell lineage. Using scRNA‐seq, the dynamic changes in cell types and their molecular functions during bladder regeneration were revealed. The single‐cell regeneration landscape of tissue‐engineered bladder included 13 cell types (53.13% nonimmune cells and 46.87% immune cells). Among them, fibroblasts, SMCs, Ens, and macrophages play important roles in bladder regeneration. Cell–cell communication across the four cell types showed that each cell type uniquely expressed receptors and ligands at each stage.

Superficial or structural modification of the scaffold material has a considerable influence on the biocompatibility and functionality of materials.^1^ Quick urothelial regeneration and differentiation are necessary to limit urine leakage and to form a functional barrier to urine, which may lead to unfavourable inflammatory responses and graft shrinkage.^25^ Blood vessel regeneration was needed to provide nutrients and oxygen and to remove waste and injured cells. SMC regeneration was essential to restore bladder compliance and contractility. Scaffold designs for bladder reconstruction have focused on compatibility and promoting the growth of these cell types, both in vitro and in vivo.^9^ PSIS is a promising biomaterial supporting the growth of urothelial cells, SMCs, endothelial cells, and neurons.^9^ However, the instability of cell development and the lack of key cells in the process of early regeneration will directly affect the success rate of tissue regeneration. Campodonico et al. reported that urothelial cells and blood vessels were distinguishable at 5 days after PSIS implantation in rabbit bladders.^26^ Su et al. found that PSIS was useful for the growth of nerve cells.^27^ In a rat study, epithelialization was observed by day 4, and smooth muscle began to regenerate 2 weeks after surgery.^28^ Neovascularity was found 2 weeks postoperatively, and neural elements formed around developing smooth muscle bundles as early as 4 weeks after grafting.^28^ Inconsistent results may indicate that previous researchers did not find the key cells guiding tissue regeneration. These factors could also explain the failure of clinical studies.^11^, ^13^, ^29^ Nevertheless, PSIS‐seeded cells may provide better outcomes.^1^, ^11^ Notably, we found that Saa3^+^ fibroblasts might mediate tissue remodelling and Notch3^+^ SMCs might modulate contractility in the current study, which are the key components for bladder morphology and functional restoration. The functions of Notch3^+^ SMCs or Saa3^+^ fibroblasts could be modified and controlled in basic studies. We believe that a better outcome could be achieved if a modified PSIS seeded Notch3^+^ SMCs or Saa3^+^ fibroblasts was applied to perform AC.

Importantly, synchronous development of all Ens was observed, suggesting that vascularization at an early stage directly affected cell migration and tissue regeneration, and vascularization with tissue orientation would play an important role in bladder remodelling.

The normal process of wound healing involves infiltration of inflammatory cells. Nine clusters of immune cells were identified in our study. They promoted cell regeneration, coordinated cell migration, and adhesion, and balanced the inflammatory response via cytokine secretion at each stage. Implantation of PSIS may initiate injuries and early pro‐inflammation reactions characterized by infiltration of neutrophils and M1 macrophages. Pro‐inflammation reactions may lead to degradation of the PSIS and drive the matrix into a fetal‐like state.^30^ In late stages, pro‐inflammatory cells fade away, and anti‐inflammatory cells emerge, which involves macrophage polarization to the M2 phenotype. These anti‐inflammatory cells interact with each other to facilitate matrix deposition and tissue reconstruction. However, dysregulation of matrix remodelling results in several pathological conditions, such as aggressive inflammation, difficult healing, and non‐functional fibrosis.^30^ Thus, proper inhibition of neutrophils and macrophages may reduce postoperative inflammation and reverse tissue fibrosis. However, more studies are warranted to explore immune involvement in the process of tissue regeneration.

One of the most challenging issues of functional bladder regeneration is innervation.^4^ Regrettably, nerve cells were not identified in the constructed landscape, suggesting that neurolization might be more difficult than vascularization. The storage function of the urinary bladder depends entirely on the autonomic nervous system. PSIS showed considerable ability to support Schwann cell adhesion, survival, migration, and proliferation on its surface when cocultured with Schwann cells,^27^ suggesting that nerve cells can be delivered during PSIS implantation. Although previous studies reported that nerve cells can be delivered during PSIS implantation, no nerve cells were captured in our study, indicating that the PSIS of composite nerve cells may be more favourable for functional bladder regeneration.

Several limitations should be noted. First, the current study was performed with a short‐term follow‐up, and the functionality of the bladder was not tested. Second, C57BL/6 mice were included for landscape visualization due to genetic stability, but it is difficult to perform urodynamic studies in the mouse bladder. Long‐term studies in patients who needed AC but failed should be considered. Third, the function of specific cells was mostly assessed based on the expression of some functional genes and GO/KEGG analyses, and more in vivo and in vitro studies are needed to validate these results.

## 5 CONCLUSION

The single‐cell remodelling landscape of tissue‐engineered bladder with PSIS included 13 cell types. Each cell type had a unique function at each stage. Major four types of cells with unique functions might underlie an unsatisfied remodelling process of bladder after PSIS. These findings may help surficial modifications of PSIS and finding a better alternative of PSIS, but more in vivo and in vitro studies are needed to validate these results.

## AUTHOR CONTRIBUTIONS

De‐yi Luo, Hong Shen, Liao Peng, Qing He, and Xi Jin designed the project; Liao Peng, Xiao‐shuai Gao, Wei Wang, and Qing He performed sample processing; Liao Peng and Qing He analysed experiments; Liao Peng performed wet laboratory experiments; Liao Peng performed computational analysis; Xi Jin and Xiao Zeng. assisted with computational analysis; Writing and editing were carried out by Liao Peng; Supervision was performed by De‐yi Luo and Hong Shen; De‐yi Luo, Xi Jin and Hong Shen obtained funding.

## CONFLICT OF INTEREST

The author(s) declared no potential conflicts of interest with respect to the research, authorship, and/or publication of this article.

## Supporting information


**Table S1.** The basic information of single‐cell RNA sequencing of 24 mice.
**Table S2.** Composition ratio of cell frequency in the four stages (corresponding to Figure S1C).
**Table S3.** Top 30 interaction pairs at each stage.
**Table S4.** The proportion of specific cell subsets at each stage.

**Figure S1.** Function analysis of specific cell types. (A) UMAP plot annotated by sample. (B) Violin plots showing the representative markers of each cluster. (C) The distribution of cells at each stage. (D) Enrichment analysis (biological process) of differentially expressed genes (DEG) from each cluster. B, B cell; DC, dendritic cell; En, endothelial cell; Ep, epithelial cell; Fib, fibroblast; MΦ, macrophage; Mast, mast cell; Mon, monocyte; Neu, neutrophil; NK, natural killer cell; Plasma, plasma cell; SMC, smooth muscle cell; T cells.

**Figure S2.** The interaction between SMCs, Fibs, Ens, and MΦs and the representative ligands and receptors of these four cell types at each stage. B, B cell; DC, dendritic cell; En, endothelial cell; Ep, epithelial cell; Fib, fibroblast; MΦ, macrophage; Mast, mast cell; Mon, monocyte; Neu, neutrophil; NK, natural killer cell; Plasma, plasma cell; SMC, smooth muscle cell; T cells.

**Figure S3.** Enrichment analysis (biological process) of the top 30 ligands and receptors at each stage.

**Figure S4.** Focused analysis of Eps and Neus. (A) Enrichment analysis (biological process) of DEGs of Eps at each stage obtained by comparison with the sham group. (B) Three clusters of Eps were identified by reclustering. Pseudotime trajectory analysis of sub‐Eps by distribution (C), pseudotime (D), and cluster (E),(F) The deceased expression of inflammatory genes over time. (G),(H) Eps‐3 response to stimulation via the HIF‐1 signalling pathway. (I) Three clusters of Neus were identified by reclustering. Pseudotime trajectory analysis of sub‐Neus by distribution (J), pseudotime (K), group (L), and cluster (M),(N) Enrichment analysis (biological process) of DEGs of Neus‐2. DEG, differently expressed gene; Neu, neutrophil.

**Figure S5.** T cell diversity. (A) Enrichment analysis (biological process) of DEGs of T cells at each stage obtained by comparison with the sham group. (B) Eight clusters of T cells were identified by reclustering. (C) The top 10 DEGs of each T‐cell subset. Pseudotime trajectory analysis of T‐cell subsets by pseudotime (D), cluster (E), group (F), and distribution (G),(H) Enrichment analysis (biological process) of DEGs of proliferating T cells. DEG, differently expressed gene.

## Data Availability

All data needed to evaluate the conclusions in the paper are present in the paper and/or the Supplementary Materials.

## References

[1] Zhao P , Li X , Fang Q , et al. Surface modification of small intestine submucosa in tissue engineering. Regener Biomater. 2020;7(4):339‐348. 10.1093/rb/rbaa014 PMC7414999 32793379

[2] Colazo JM , Evans BC , Farinas AF , Al‐Kassis S , Duvall CL , Thayer WP . Applied bioengineering in tissue reconstruction, replacement, and regeneration. Tissue Eng Part B Rev. 2019;25(4):259‐290. 30896342 10.1089/ten.teb.2018.0325 PMC6686706

[3] Abdulghani S , Mitchell GR . Biomaterials for in situ tissue regeneration: a review. Biomolecules. 2019;9(11):750. 31752393 10.3390/biom9110750 PMC6920773

[4] Adamowicz J , Kowalczyk T , Drewa T . Tissue engineering of urinary bladder ‐ current state of art and future perspectives. Cent Eur J Urol. 2013;66(2):202‐206. 10.5173/ceju.2013.02.art23 PMC3936152 24579029

[5] Oberpenning F , Meng J , Yoo JJ , Atala A . De novo reconstitution of a functional mammalian urinary bladder by tissue engineering. Nat Biotechnol. 1999;17(2):149‐155. 10052350 10.1038/6146

[6] Lin HK , Godiwalla SY , Palmer B , et al. Understanding roles of porcine small intestinal submucosa in urinary bladder regeneration: identification of variable regenerative characteristics of small intestinal submucosa. Tissue Eng Part B Rev. 2014;20(1):73‐83. 23777420 10.1089/ten.teb.2013.0126 PMC3922139

[7] Ginsberg DA , Boone TB , Cameron AP , et al. The AUA/SUFU guideline on adult neurogenic lower urinary tract dysfunction: treatment and follow‐up. J Urol. 2021;206(5):1106‐1113. 34495688 10.1097/JU.0000000000002239

[8] Cheng PJ , Myers JB . Augmentation cystoplasty in the patient with neurogenic bladder. World J Urol. 2020;38(12):3035‐3046. 31511969 10.1007/s00345-019-02919-z

[9] Lin HK , Madihally SV , Palmer B , Frimberger D , Fung KM , Kropp BP . Biomatrices for bladder reconstruction. Adv Drug Deliv Rev. 2015;82‐83:47‐63. 10.1016/j.addr.2014.11.020 25477305

[10] Lam Van Ba O , Aharony S , Loutochin O , Corcos J . Bladder tissue engineering: a literature review. Adv Drug Deliv Rev. 2015;82‐83:31‐37. 10.1016/j.addr.2014.11.013 25446136

[11] Atala A , Bauer SB , Soker S , Yoo JJ , Retik AB . Tissue‐engineered autologous bladders for patients needing cystoplasty. Lancet (London, England). 2006;367(9518):1241‐1246. 16631879 10.1016/S0140-6736(06)68438-9

[12] Kropp BP . Small‐intestinal submucosa for bladder augmentation: a review of preclinical studies. World J Urol. 1998;16(4):262‐267. 9775425 10.1007/s003450050064

[13] Schaefer M , Kaiser A , Stehr M , Beyer HJ . Bladder augmentation with small intestinal submucosa leads to unsatisfactory long‐term results. J Pediatr Urol. 2013;9(6 Pt A):878‐883. 23332207 10.1016/j.jpurol.2012.12.001

[14] Zhang F , Liao L . Long‐term follow‐up of neurogenic bladder patients after bladder augmentation with small intestinal submucosa. World J Urol. 2020;38(9):2279‐2288. 31712957 10.1007/s00345-019-03008-x

[15] Zheng GX , Terry JM , Belgrader P , et al. Massively parallel digital transcriptional profiling of single cells. Nat Commun. 2017;8:14049. 28091601 10.1038/ncomms14049 PMC5241818

[16] Kropp BP , Eppley BL , Prevel CD , et al. Experimental assessment of small intestinal submucosa as a bladder wall substitute. Urology. 1995;46(3):396‐400. 7660517 10.1016/S0090-4295(99)80227-1

[17] Tu DD , Seth A , Gil ES , Kaplan DL , Mauney JR , Estrada CR Jr . Evaluation of biomaterials for bladder augmentation using cystometric analyses in various rodent models. J Visualized Exp. 2012;66:3981. 10.3791/3981 PMC3486757 22907252

[18] He S , Wang LH , Liu Y , et al. Single‐cell transcriptome profiling of an adult human cell atlas of 15 major organs. Genome Biol. 2020;21(1):294. 33287869 10.1186/s13059-020-02210-0 PMC7720616

[19] Yu Z , Liao J , Chen Y , et al. Single‐cell transcriptomic map of the human and mouse bladders. J Am Soc Nephrol. 2019;30(11):2159‐2176. 31462402 10.1681/ASN.2019040335 PMC6830796

[20] Efremova M , Vento‐Tormo M , Teichmann SA , Vento‐Tormo R . CellPhoneDB: inferring cell‐cell communication from combined expression of multi‐subunit ligand‐receptor complexes. Nat Protoc. 2020;15(4):1484‐1506. 32103204 10.1038/s41596-020-0292-x

[21] Trapnell C , Cacchiarelli D , Grimsby J , et al. The dynamics and regulators of cell fate decisions are revealed by pseudotemporal ordering of single cells. Nat Biotechnol. 2014;32(4):381‐386. 24658644 10.1038/nbt.2859 PMC4122333

[22] Goldman JA , Poss KD . Gene regulatory programmes of tissue regeneration. Nat Rev Genet. 2020;21(9):511‐525. 32504079 10.1038/s41576-020-0239-7 PMC7448550

[23] Tardito S , Martinelli G , Soldano S , et al. Macrophage M1/M2 polarization and rheumatoid arthritis: a systematic review. Autoimmun Rev. 2019;18(11):102397. 31520798 10.1016/j.autrev.2019.102397

[24] Taciak B , Pruszynska I , Kiraga L , Bialasek M , Krol M . Wnt signaling pathway in development and cancer. J Physiol Pharmacol. 2018;69(2). doi:10.26402/jpp.2018.2.07 29980141

[25] Sievert KD , Tanagho EA . Organ‐specific acellular matrix for reconstruction of the urinary tract. World J Urol. 2000;18(1):19‐25. 10766039 10.1007/s003450050004

[26] Campodonico F , Benelli R , Michelazzi A , Ognio E , Toncini C , Maffezzini M . Bladder cell culture on small intestinal submucosa as bioscaffold: experimental study on engineered urothelial grafts. Eur Urol. 2004;46(4):531‐537. 15363573 10.1016/j.eururo.2004.04.019

[27] Su Y , Zeng BF , Zhang CQ , Zhang KG , Xie XT . Study of biocompatibility of small intestinal submucosa (SIS) with Schwann cells in vitro. Brain Res. 2007;1145:41‐47. 17367764 10.1016/j.brainres.2007.01.138

[28] Sutherland RS , Baskin LS , Hayward SW , Cunha GR . Regeneration of bladder urothelium, smooth muscle, blood vessels and nerves into an acellular tissue matrix. J Urol. 1996;156(2 Pt 2):571‐577. 8683736 10.1097/00005392-199608001-00002

[29] Zhang F , Liao L . Tissue engineered cystoplasty augmentation for treatment of neurogenic bladder using small intestinal submucosa: an exploratory study. J Urol. 2014;192(2):544‐550. 24681330 10.1016/j.juro.2014.01.116

[30] Xu M , Su T , Jin X , et al. Inflammation‐mediated matrix remodeling of extracellular matrix‐mimicking biomaterials in tissue engineering and regenerative medicine. Acta Biomater. 2022;S1742‐7061(22)00492‐5. doi:10.1016/j.actbio.2022.08.015 35970482

