# Supplementary material for: Remodelling landscape of tissue‐engineered bladder with porcine small intestine submucosa using single‐cell RNA sequencing
Source: Cell Prolif. 2022 Sep 30;56(1):e13343. doi: 10.1111/cpr.13343 (PMC9816928; doi:10.1111/cpr.13343)
Supplement: Supplementary file 1 — Table S1. The basic information of single‐cell RNA sequencing of 24 mice. Table S2. Composition ratio of cell frequency in the four stages (corresponding to Figure S1C). Table S3. Top 30 interaction pairs at each stage. Table S4. The proportion of specific cell subsets at each stage. [file CPR-56-e13343-s006.docx]

| **Supplementary Table 1** The basic information of single-cell RNA sequencing of 24 mice. | | | | |
| --- | --- | --- | --- | --- |
| Items | Sham (n=6) | Week 1 (n=6) | Week 3 (n=6) | Week 6 (n=6) |
| Estimated Number of Cells | 15,678 | 9,843 | 4,993 | 8,176 |
| Mean Reads per Cell | 50,720 | 65,911 | 67,409 | 61,394 |
| Median Genes per Cell | 2,368 | 1,838 | 1,010 | 1,433 |
| Number of Reads | 795,191,423 | 648,763,864 | 336,575,504 | 501,959,053 |
| Valid Barcodes | 97.80% | 97.10% | 97.20% | 97.80% |
| Sequencing Saturation | 74.80% | 66.70% | 82.50% | 81.70% |
| Q30 Bases in Barcode | 95.50% | 95.30% | 95.30% | 95.30% |
| Q30 Bases in RNA Read | 92.20% | 93.30% | 91.60% | 91.20% |
| Q30 Bases in UMI | 94.60% | 93.50% | 92.20% | 94.60% |
| Reads Mapped to Genome | 94.60% | 93.10% | 84.70% | 82.40% |
| Reads Mapped Confidently to Genome | 85.20% | 82.80% | 75.60% | 72.60% |
| Reads Mapped Confidently to Intergenic Regions | 2.10% | 2.60% | 5.10% | 4.40% |
| Reads Mapped Confidently to Intronic Regions | 19.70% | 17.70% | 24.50% | 22.80% |
| Reads Mapped Confidently to Exonic Regions | 63.40% | 62.50% | 46.10% | 45.50% |
| Reads Mapped Confidently to Transcriptome | 59.40% | 59.00% | 43.30% | 43.10% |
| Reads Mapped Antisense to Gene | 1.90% | 1.60% | 1.40% | 1.00% |
| Fraction Reads in Cells | 92.90% | 93.70% | 91.50% | 90.50% |
| Total Genes Detected | 29,591 | 29,104 | 25,553 | 27,806 |
| Median UMI Counts per Cell | 6,305 | 5,257 | 2,497 | 2,669 |
| UMI, unique molecular identifiers |  |  |  |  |

| **Supplementary Table 2** Composition ratio of cell frequency in the four stages (corresponding to Figure S1C). | | | | | |
| --- | --- | --- | --- | --- | --- |
|  | Total | Week 1 (n=6) | Week 3 (n=6) | Week 6 (n=6) | Sham (n=6) |
| Fib | 15689 | 2111 (13.46%) | 2058 (13.12%) | 4786 (30.51%) | 6734 (42.92%) |
| SMC | 2077 | 5 (0.24%) | 50 (2.41%) | 577 (27.78%) | 1445 (69.57%) |
| En | 793 | 96 (12.11%) | 129 (16.27%) | 510 (64.31%) | 58 (7.31%) |
| Ep | 461 | 255 (55.31%) | 9 (1.95%) | 72 (15.62%) | 125 (27.11%) |
| Neu | 6128 | 3710 (60.54%) | 2236 (36.49%) | 127 (2.07%) | 55 (0.90%) |
| T | 4620 | 2132 (46.15%) | 893 (19.33%) | 1222 (26.45%) | 373 (8.07%) |
| DC | 2630 | 412 (15.67%) | 161 (6.12%) | 718 (27.30%) | 1339 (50.91%) |
| MΦ | 2152 | 1412 (65.61%) | 330 (15.33%) | 270 (12.55%) | 140 (6.51%) |
| Mon | 430 | 193 (44.88%) | 0 (0.00%) | 93 (21.63%) | 144 (33.49%) |
| NK | 245 | 161 (65.71%) | 84 (34.29%) | 0 (0.00%) | 0 (0.00%) |
| Plasma | 234 | 8 (3.42%) | 9 (3.85%) | 181 (77.35%) | 36 (15.38%) |
| B | 173 | 64 (36.99%) | 100 (57.80%) | 4 (2.31%) | 5 (2.89%) |
| Mast | 170 | 44 (25.88%) | 45 (26.47%) | 57 (33.53%) | 24 (14.12%) |
| Fib, fibroblasts; SMC, smooth muscle cell; En, endothelial cell; Ep, epithelial cell; Neu, neutrophil; T, T cell; DC, dendric cell; MΦ, macrophage; Mon, monocyte; NK, natural killer cell; Plasma, plasma cell; B, B cell; Mast, mast cell. | | | | | |

| **Supplementary Table 3** Top 30 interaction pairs at each stage. | | | |
| --- | --- | --- | --- |
| Week 1 | Week 3 | Week 6 | Sham |
| Wnt11-Klrg2  Epha3-Efna4  Epha1-Efna4  Erbb3-Nrg2  Efna2-Epha1  Cd70-Tnfrsf17  Col8a2-A10b1  Efna4-Epha7  Efna2-Epha7  Bmp7-Bmpr1b/Bmpr2  Bmp7-Bmr1b/Avr2a  Nppc-Npr2  Bmp7-Bmr1b/Avr2b  Bmp5-Bmr1b/Avr2a  Bmp5-Bmr1b/Avr2b  Bmp6-Bmpr1b/Bmpr2  Bmp6-Bmr1b-Avr2a  Bmp6-Bmr1b-Avr2b  Wnt2-Fzd9  Gdf6-Bmpr1b/Bmpr2  Gdf6-Bmr1b/Avr2a  Gdf6-Bmr1b/Avr2b  Plexina3-Sema3a  Bmp5-Bmpr1b/Bmpr2  Xcl2-Xcr1  Efna2-Epha3  Cadm3-Cadm1  Col16a1-A10b1  Col14a1-A10b1  Eda-Eda2r | Col20a1-A11b1  Xcl2-Xcr1  Tnfsf10-Tnfrsf11b  Bmp7-Slamf1  Ptn-Ptprz1  Plexina4-Sema3a  Plexina3-Sema3a  Ccl25-Ccr9  Pgf-Flt1  Gdf11-Acvr-1b2b  Col24a1-A10b1  Col20a1-A10b1  Col17a1-A10b1  Notch4-Jag2  Notch4-Dll4  Col24a1-A11b1  Bmp7-Bmr1b/Avr2b  Bmp7-Bmr1a/Avr2b  Col17a1-A11b1  Bmp7-Acvr-1a2b  Tnfsf8-Tnfrsf8  Camp-Fpr2  Fcer2-Cr2  Notch4-Dll1  Cadm1-Cadm1  Gdf11-Tgfr/Avr2b  Pthlh-Pth1r  Btc-Erbb3  Btc-Egfr  Bmp6-Acvr-1a2b | Plexina4-Sema3a  Ccl8-Ccr3  Fcer2-Cr2  Dsc2-Dsg2  Vegfd-Flt4  Ccl11-Dpp4  Vegfc-Kdr  Col24a1-A11b1  Vegfc-Flt4  Ccl11-Ccr3  Lgr4-Nrg1  Ccl24-Ccr3  Notch3-Jag2  Pthlh-Pth1r  Fgfr2-Fgfr3  Ccl7-Ccr3  Cspg4-A3b1  Col27a1-A11b1  Notch3-Dll4  Notch3-Dll1  Ccl21-Ccr7  Ptprr-Fgfr2  Tnfsf10-Tnfrsf10b  Plexina1-Sema3a  Cxcl1-Cxcr1  Epha4-Fgfr2  Epha4-Fgfr3  Vegfd-Kdr  Col11a1-A11b1  Efna1-Epha4 | Pthlh-Pth1r  Thbs1-A3b1  Efna1-Epha3  Vegfd-Kdr  Efna1-Epha4  Il2-Il2ra  Bmp7-Bmpr1b/Bmpr2  Tnfsf10-Tnfrsf10b  Bmp7-Bmr1b/Avr2a  Bmp5-Acvr-1a2a  Efna1-Epha7  Bmp5-Bmpr1b/Bmpr2-  Bmp5-Bmr1a/Acr2a  Ccl7-Ccr10  Bmp5-Bmr1b/Avr2a  Bmp6-Bmr1b/Avr2a  Ccl21-Ccr7  Inhbb-Acvr-1a2a  Ccl21-Ackr4  Ccl11-Ackr4  Pdcd1-Cd274  Ccl5-Ackr4  Pdcd1-Pdcd1lg2  Vegfd-Flt4  Bdnf-Sort1  Pgf-Flt1  Plexina3-Sema3a  TNFSF9-PVR  FGF2-CD44  TGFB1-TGFBR3 |

| **Supplementary Table 4** The proportion of specific cell subsets at each stage. | | | | |
| --- | --- | --- | --- | --- |
|  | Sham | Week1 | Week2 | Week3 |
|  |  |  |  |  |
| **The proportion of fibroblast cell subsets in each stage** | | | | |
| Fib 1 | 41.43% | 5.73% | 49.28% | 35.21% |
| Fib 2 | 13.09% | 2.45% | 8.96% | 17.13% |
| Fib 3 | 11.14% | 11.78% | 16.67% | 15.89% |
| Fib 4 | 11.87% | 20.29% | 7.71% | 8.73% |
| Fib 5 | 9.05% | 6.06% | 4.30% | 12.90% |
| Fib 6 | 6.88% | 0.49% | 5.73% | 4.14% |
| Fib 7 | 2.43% | 51.88% | 2.15% | 2.43% |
| Fib 8 | 3.60% | 0.00% | 4.30% | 3.20% |
| Fib 9 | 0.51% | 1.31% | 0.90% | 0.37% |
|  |  |  |  |  |
| **The proportion of SMC subsets in each stage** | | | | |
| SMC 1 | 60.76% | 0.00% | 10.00% | 47.49% |
| SMC 2 | 30.87% | 0.00% | 8.00% | 27.21% |
| SMC 3 | 5.05% | 100.00% | 58.00% | 20.62% |
| SMC 4 | 2.98% | 0.00% | 10.00% | 2.95% |
| SMC 5 | 0.35% | 0.00% | 14.00% | 1.73% |
|  |  |  |  |  |
| **The proportion of endothelial cell (EC) subsets in each stage** | | | | |
| Vein ECs | 38.78% | 69.79% | 82.03% | 71.02% |
| Lymphatic ECs | 57.14% | 3.13% | 5.47% | 20.20% |
| Artery ECs | 4.08% | 27.08% | 12.50% | 8.78% |
|  |  |  |  |  |
| **The proportion of macrophage (MΦ) subsets in each stage** | | | | |
| MΦ 1 | 1.43% | 52.41% | 37.58% | 13.33% |
| MΦ 2 | 92.86% | 39.38% | 61.21% | 84.44% |
| MΦ 3 | 5.71% | 8.22% | 1.21% | 2.22% |
|  |  |  |  |  |
| **The proportion of epithelial cell subsets in each stage** | | | | |
| Ep 1 | 73.60% | 31.76% | 33.33% | 65.28% |
| Ep 2 | 26.40% | 46.67% | 22.22% | 34.72% |
| Ep 3 | 0.00% | 21.57% | 44.44% | 0.00% |
|  |  |  |  |  |
| **The proportion of neutrophil subsets in each stage** | | | | |
| Neu 1 | 5.45% | 41.91% | 38.51% | 19.69% |
| Neu 2 | 1.82% | 30.49% | 29.20% | 6.30% |
| Neu 3 | 92.73% | 25.82% | 27.86% | 70.08% |
| Neu 4 | 0.00% | 1.78% | 4.43% | 3.94% |
|  |  |  |  |  |
| **The proportion of T cell subsets in each stage** | | | | |
| CD4^+^ exhausted T cell | 6.49% | 31.12% | 26.10% | 23.41% |
| Cd8^+^ effector T cell | 1.35% | 33.82% | 18.03% | 3.79% |
| Gamma delta T | 26.49% | 4.50% | 21.71% | 17.64% |
| Innate lymphocytes | 53.24% | 0.40% | 2.97% | 13.93% |
| Proliferation T | 3.51% | 16.61% | 1.42% | 3.22% |
| Central memory T cell | 1.62% | 5.95% | 8.90% | 3.46% |
| Regulatory T cell | 1.35% | 1.45% | 3.91% | 3.63% |
| Unknown | 5.95% | 6.15% | 16.96% | 30.92% |
