# Supplementary figures and images for: Remodelling landscape of tissue‐engineered bladder with porcine small intestine submucosa using single‐cell RNA sequencing
Source: Cell Prolif. 2022 Sep 30;56(1):e13343. doi: 10.1111/cpr.13343 (PMC9816928; doi:10.1111/cpr.13343)

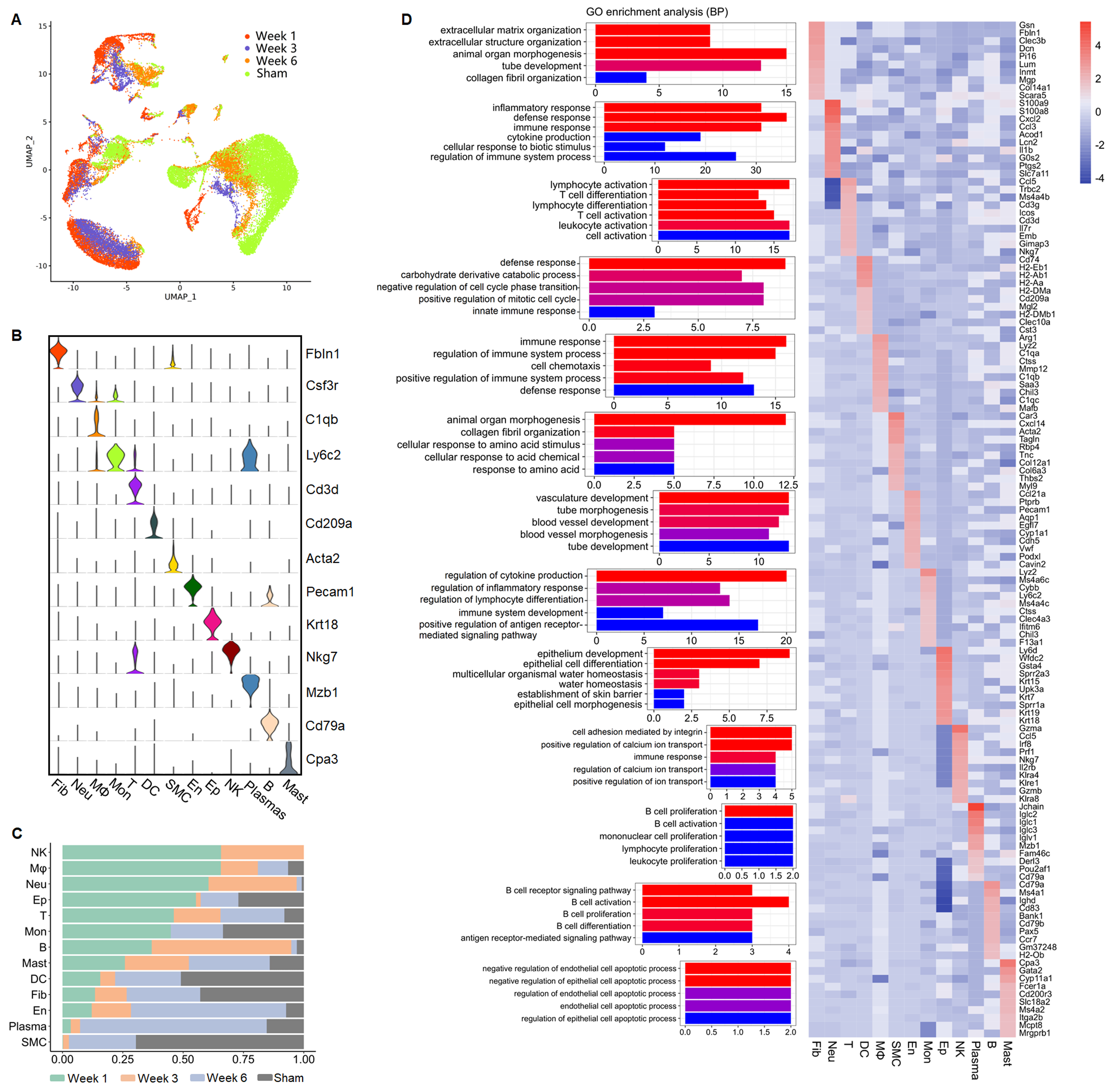

Supplement: Supplementary file 2 — Figure S1. Function analysis of specific cell types. (A) UMAP plot annotated by sample. (B) Violin plots showing the representative markers of each cluster. (C) The distribution of cells at each stage. (D) Enrichment analysis (biological process) of differentially expressed genes (DEG) from each cluster. B, B cell; DC, dendritic cell; En, endothelial cell; Ep, epithelial cell; Fib, fibroblast; MΦ, macrophage; Mast, mast cell; Mon, monocyte; Neu, neutrophil; NK, natural killer cell; Plasma, plasma cell; SMC, smooth muscle cell; T cells. [file CPR-56-e13343-s002.png]

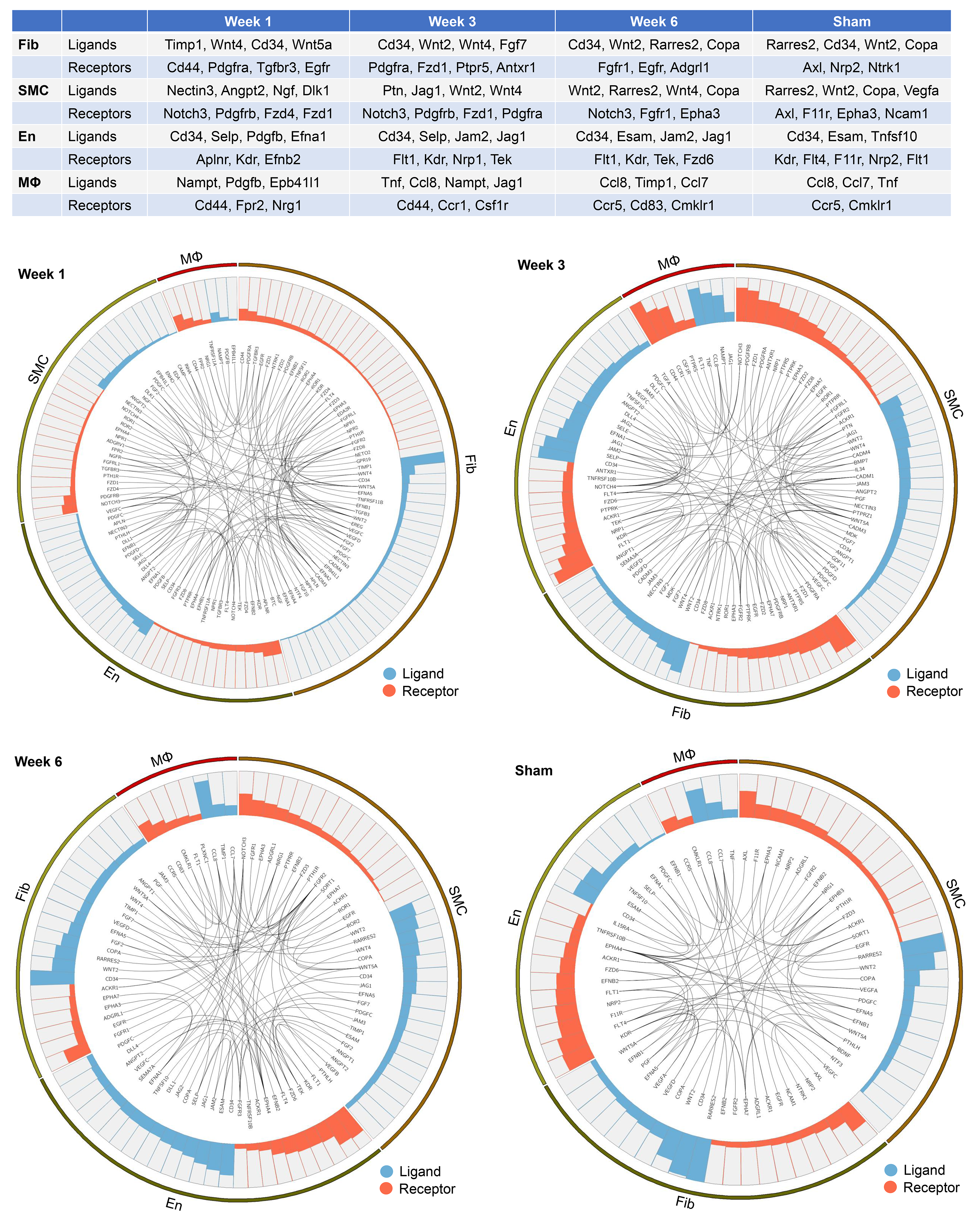

Supplement: Supplementary file 3 — Figure S2. The interaction between SMCs, Fibs, Ens, and MΦs and the representative ligands and receptors of these four cell types at each stage. B, B cell; DC, dendritic cell; En, endothelial cell; Ep, epithelial cell; Fib, fibroblast; MΦ, macrophage; Mast, mast cell; Mon, monocyte; Neu, neutrophil; NK, natural killer cell; Plasma, plasma cell; SMC, smooth muscle cell; T cells. [file CPR-56-e13343-s005.png]

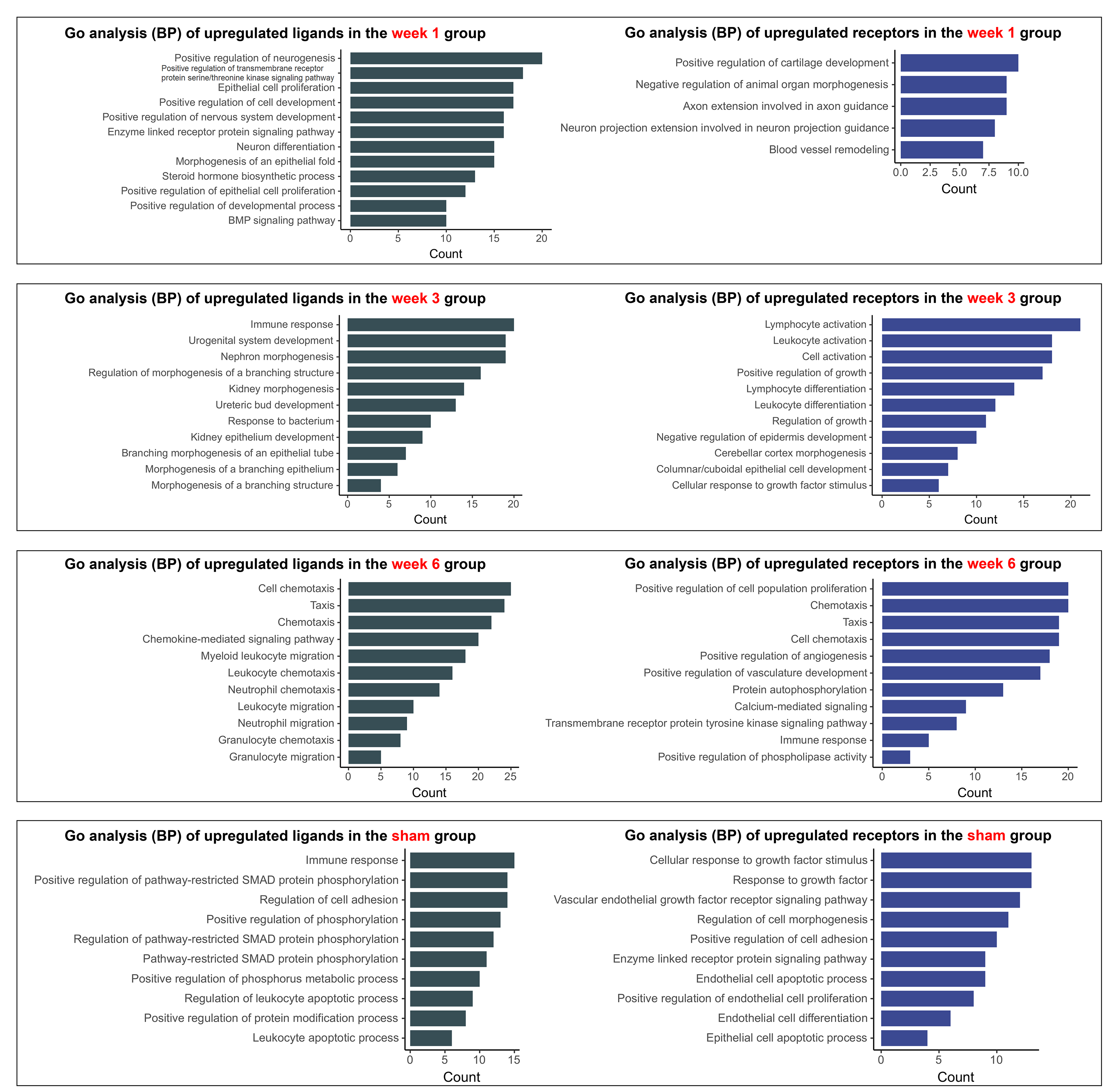

Supplement: Supplementary file 4 — Figure S3. Enrichment analysis (biological process) of the top 30 ligands and receptors at each stage. [file CPR-56-e13343-s004.png]

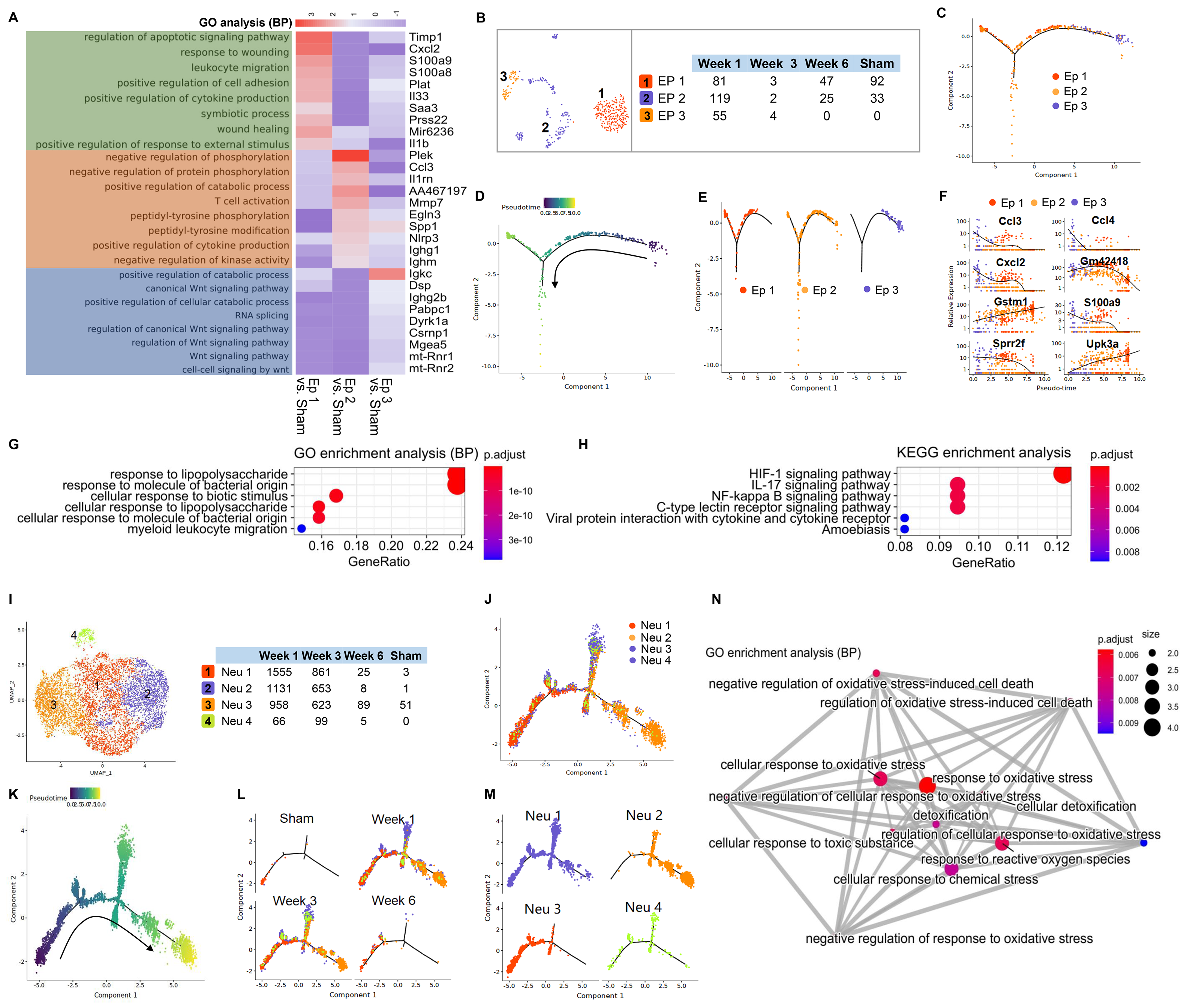

Supplement: Supplementary file 5 — Figure S4. Focused analysis of Eps and Neus. (A) Enrichment analysis (biological process) of DEGs of Eps at each stage obtained by comparison with the sham group. (B) Three clusters of Eps were identified by reclustering. Pseudotime trajectory analysis of sub‐Eps by distribution (C), pseudotime (D), and cluster (E),(F) The deceased expression of inflammatory genes over time. (G),(H) Eps‐3 response to stimulation via the HIF‐1 signalling pathway. (I) Three clusters of Neus were identified by reclustering. Pseudotime trajectory analysis of sub‐Neus by distribution (J), pseudotime (K), group (L), and cluster (M),(N) Enrichment analysis (biological process) of DEGs of Neus‐2. DEG, differently expressed gene; Neu, neutrophil. [file CPR-56-e13343-s003.png]

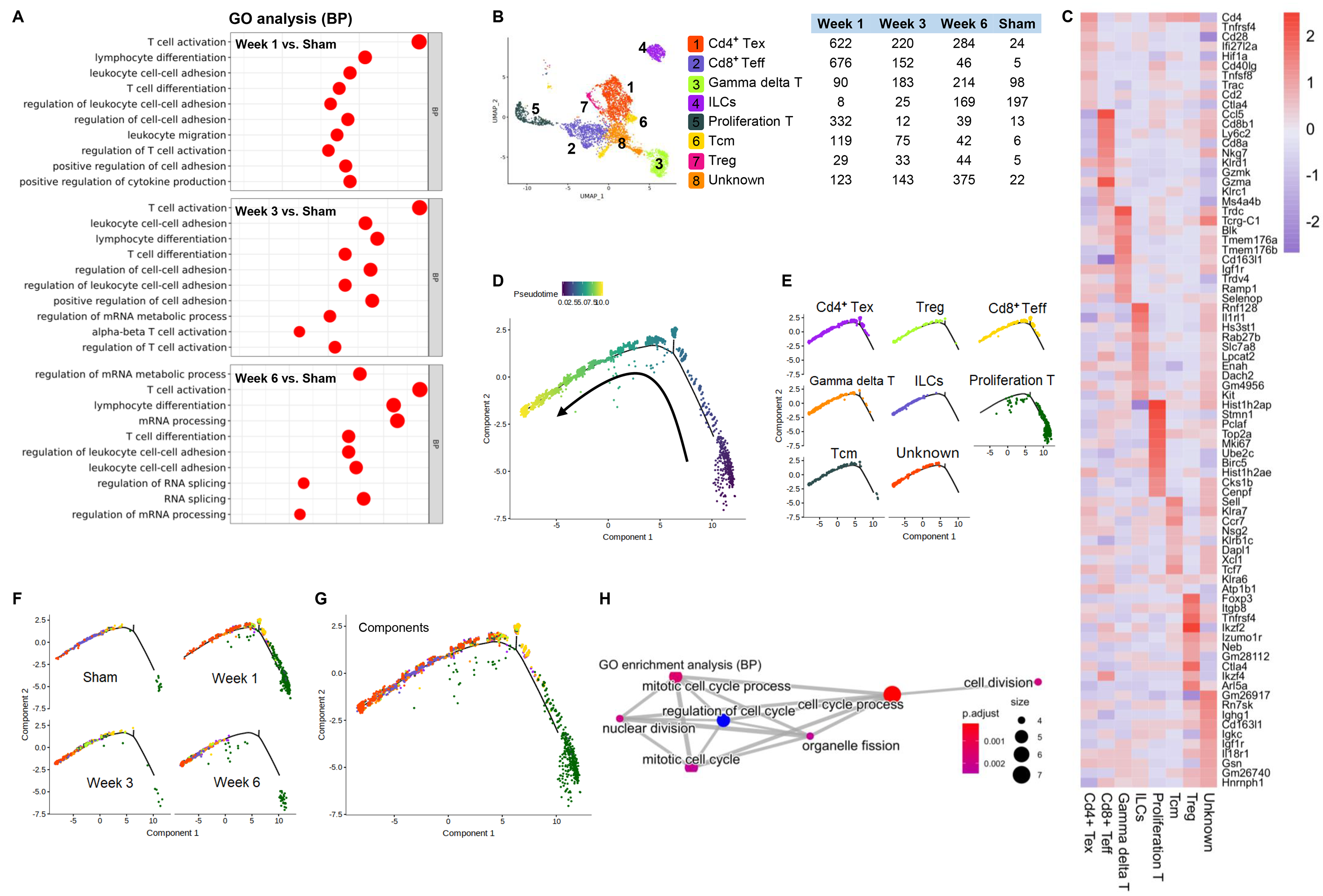

Supplement: Supplementary file 6 — Figure S5. T cell diversity. (A) Enrichment analysis (biological process) of DEGs of T cells at each stage obtained by comparison with the sham group. (B) Eight clusters of T cells were identified by reclustering. (C) The top 10 DEGs of each T‐cell subset. Pseudotime trajectory analysis of T‐cell subsets by pseudotime (D), cluster (E), group (F), and distribution (G),(H) Enrichment analysis (biological process) of DEGs of proliferating T cells. DEG, differently expressed gene. [file CPR-56-e13343-s001.png]
